# Associations of oxidative balance score with total abdominal fat mass and visceral adipose tissue mass percentages among young and middle-aged adults: findings from NHANES 2011–2018

**DOI:** 10.3389/fnut.2023.1306428

**Published:** 2023-12-05

**Authors:** Kai Wang, Minggang Deng, Jinyi Wu, Lingli Luo, Rui Chen, Fang Liu, Jiaqi Nie, Fengxi Tao, Qingwen Li, Xin Luo, Fang Xia

**Affiliations:** ^1^Department of Public Health, Wuhan Fourth Hospital, Wuhan, China; ^2^Department of Psychiatry, Wuhan Mental Health Center, Wuhan, China; ^3^Department of Psychiatry, Wuhan Hospital for Psychotherapy, Wuhan, China; ^4^Department of Pathophysiology, School of Basic Medicine and Tongji Medical College, Huazhong University of Science and Technology, Wuhan, China; ^5^School of Public Health, Wuhan University, Wuhan, China; ^6^Department of Health Promotion, XiaoGan Center for Disease Control and Pervention, Xiaogan, China

**Keywords:** abdominal adiposity, oxidative balance score, total abdominal fat mass, visceral adipose tissue mass, visceral fat accumulation

## Abstract

**Objective:**

This study aimed to explore the association of the oxidative balance score (OBS) with total abdominal fat mass (TAFM) and visceral adipose tissue mass (VATM) percentages among young and middle-aged U.S. adults.

**Methods:**

Young and middle-aged adults in the National Health and Nutrition Examination Survey (NHANES) from 2011 to 2018 were included. Analysis of variance and Rao-Scott adjusted chi-square tests were used to compare the characteristics across quartiles of OBS. Univariate and multivariate weighted logistic regression models were employed to explore the relationship between OBS and the risks of high TAFM or high VATM percentage in the general population and subgroups, while the interaction effects were tested with a likelihood test. Weighted restricted cubic spline analyses were utilized to assess the non-linear association of OBS with TAFM and VATM percentages.

**Results:**

The final sample included 8,734 young and middle-aged non-institutionalized U.S. adults representing 134.7 million adults. Compared with adults in the first quartile of OBS, those with higher OBS were less likely to have a high TAFM percentage; the ORs and 95% CI for adults in the second, third, and highest quartiles of OBS were 0.70 (0.53–0.94), 0.49 (0.36–0.60), and 0.25 (0.18–0.36), respectively. Similar trends were observed in the association between OBS and VATM percentages. Moreover, similar effects were confirmed in the sensitivity analyses and subgroup analyses according to demographic characteristics. Regarding the OBS subclass, higher dietary OBS and lifestyle OBS were also correlated with decreased ORs of high TAFM and VATM percentages.

**Conclusion:**

This study strongly suggests that higher OBS, as well as higher dietary OBS and lifestyle OBS, are significantly correlated with lower risks of abdominal obesity and visceral fat accumulation. The findings highlight the importance of an antioxidant-rich diet and maintaining a healthy lifestyle in reducing the risks.

## 1 Introduction

The obesity health issue, usually assessed by body mass index (BMI), continues its relentless advance globally, the prevalence of which has climbed from 3 to 11% among men and from 6 to 15% among women over the past 40 years (1). BMI values continued to rise until they crossed the threshold of normality and reached an average BMI of 27.8 in 2014 in the United States (2). Age-standardized prevalence of obesity and severe obesity in U.S. adults increased from 33.7 and 5.7% in 2007–2008 to 39.6 and 7.7% in 2015–2016, which has seen a significant increase over the years (3).

Obesity, gradually regarded as a chronic relapsing disease process, enlarges fat cells, and ectopic fat produces and secretes a variety of metabolic, hormonal, and inflammatory substances that are pernicious to organs, especially the liver and pancreas (4). Epidemiological studies have illustrated obesity as a significant risk factor for a variety of non-communicable chronic diseases, including diabetes, cardiovascular disease, gout, non-alcoholic fatty liver disease, and a set of cancers (5–10). Moreover, obesity could also result in a series of skeletal muscle disorders, probably on account of adipose tissue inflammation dominating skeletal muscle inflammation (11, 12). In addition to its impact on individual health, obesity also has significant economic consequences, particularly in terms of healthcare costs. The Global Burden of Disease group has estimated that elevated BMI values contributed to 4 million deaths in 2015, two-thirds of which were responsible for cardiovascular disease (13). In addition, Haijiang Dai et al. have estimated that the global number of disability-adjusted life years related to high BMI has more than doubled for both sexes between 1990 and 2017 (14).

Oxidative stress is defined as an imbalance between oxidants and antioxidants in favor of the oxidants, leading to a disruption of redox signaling and control and/or molecular damage (15). Nevertheless, it is impossible to reflect oxidative stress level by a single factor, and oxidative balance score (OBS) emerged at the time required. In addition, the exact index, with its higher simplicity and understandability, greatly improves the ability to identify individual oxidative stress levels. OBS, combining various dietary and lifestyle pro-oxidants and antioxidants, is provided to measure individual exposures to antioxidants and pro-oxidants as well as the balance, in which higher OBS indicate higher exposure to antioxidants and lower exposure to pro-oxidants (16). Moreover, OBS has been proven by Lingling Song et al. to be correlated with oxidative stress in two NHANES studies (17, 18). A number of epidemiological studies have explored the relationship between OBS and diabetes, non-alcoholic fatty liver disease, periodontitis, lung health, and vascular endothelial function (19–24). In addition, the molecular mechanisms underlying oxidative stress in relation to obesity have been widely acknowledged, such as altering regulatory factors of mitochondrial activity, promoting lipogenesis, stimulating the differentiation of preadipocytes into mature adipocytes, and regulating appetite-related hypothalamic neuron energy balance (25).

BMI, an inadequate indicator of obesity, fails to differentiate between fat mass, fat-free mass, and distribution of adipose tissue. In addition to BMI, total abdominal fat mass (TAFM) and visceral adipose tissue mass (VATM) are important indicators of abdominal obesity and visceral fat accumulation. Meanwhile, differences persist between different adipose tissues, including anatomical, cellular, molecular, physiological, clinical, and prognostic differences, leading to an elevated risk of diabetes and cardiovascular diseases in abdominal obesity compared to that in peripheral or gluteofemoral obesity (26). To the best of our knowledge, there is a lack of studies investigating the relationship between OBS and obesity. Furthermore, there is a dearth of studies that have assessed the associations between OBS and abdominal obesity, or visceral fat accumulation measured by whole-body fat distribution. Therefore, this study aimed to explore the associations between OBS and TAFM and VATM percentages in U.S. adults using the National Health and Nutrition Examination Survey (NHANES).

## 2 Materials and methods

### 2.1 Study populations

NHANES is a consecutive, population-based survey conducted by the National Center for Health Statistics of the Centers for Disease Control and Prevention that assesses nutrition and health status collected every 2 years in the U.S. population. NHANES consists of demographics, dietary, examination, laboratory, and questionnaire data, providing detailed information about demographic characteristics, socioeconomic status, physiological measurements, biochemical indicators, and standardized questionnaires about health in various aspects (27). To make the survey representative of the national population, a complex, multistage, probability sampling design with oversampling of different subpopulations is adopted, and corresponding weights are generated. Furthermore, the compensation for participants enables NHANES to collect reliable and high-quality data, which ensures the accuracy and validity of the information.^1^

**Figure 1:**
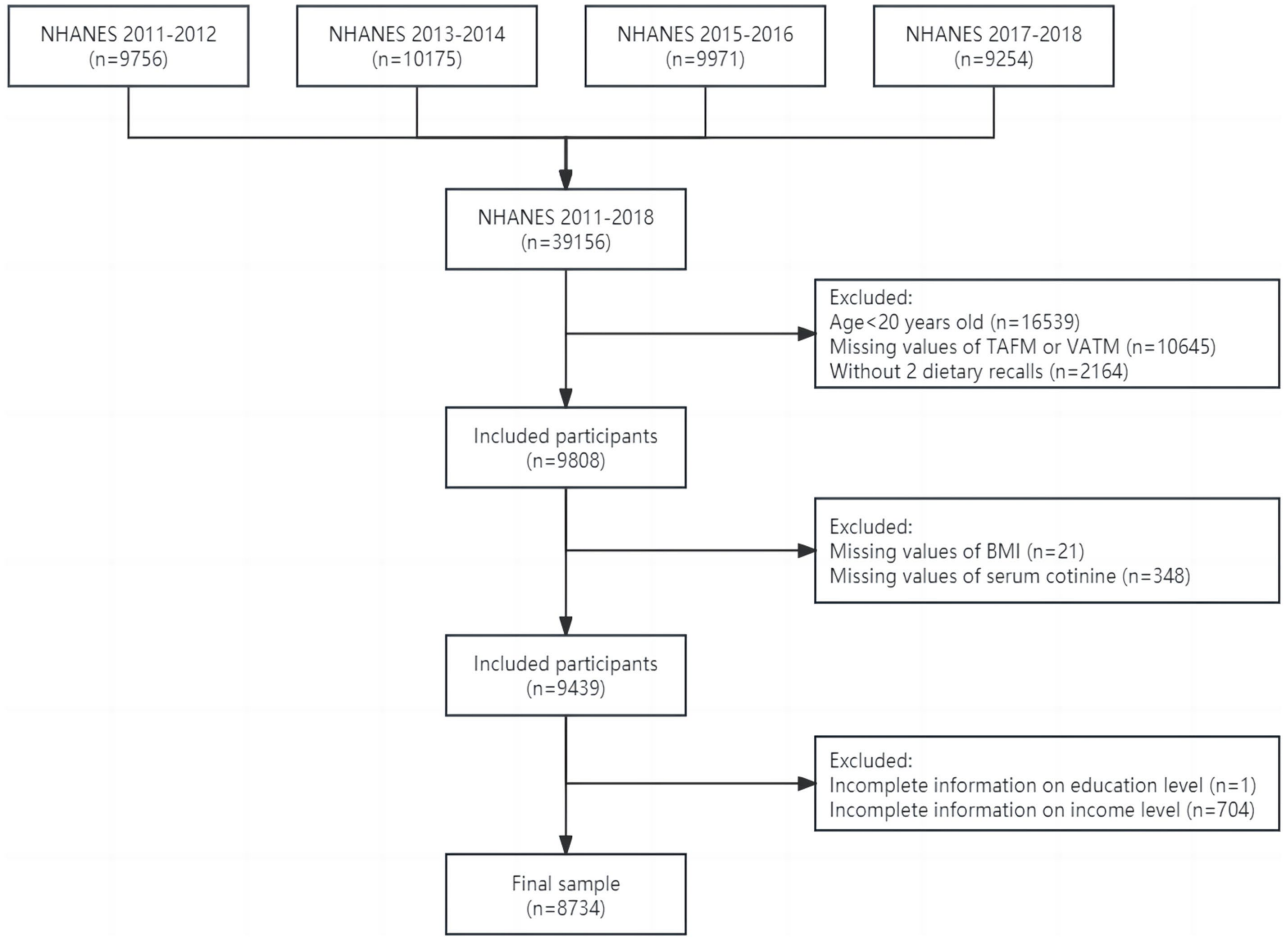
Flowchart of study design and exclusion criteria. BMI, body mass index; NHANES, National Health and Nutrition Examination Survey; TAFM, total abdominal fat mass; VATM, visceral adipose tissue mass.

To carry out this study, we included adults aged 20 years and older who had complete information about TAFM, VATM, and OBS in four cycles from NHANES 2011–2018. Details of the current study’s sampling and exclusion criteria are described in Figure 1. A total of 39,156 participants were initially included, of which 16,539 were aged less than 20 years old. After excluding participants with missing values of TAFM or VATM (*n* = 10,645), without two dietary recalls (*n* = 2,164), with missing values of BMI (*n* = 21), serum cotinine (*n* = 348), education level (*n* = 1), and family income level (*n* = 704), the final sample included 8,734 adults. All participants in NHANES provided written informed consent, and only publicly available data were used in the current analysis, so no ethical approval was needed in this study.

### 2.2 Outcome ascertainment

TAFM and VATM were measured using dual-energy x-ray absorptiometry in participants aged 8–59 years old, which was the most widely accepted method to measure body composition due in part to its speed, simplicity, and low radiation exposure (28). Participants underwent whole-body dual-energy x-ray scans in the NHANES mobile examination center, in which visceral adipose tissue and subcutaneous adipose tissue were defined by the Hologic APEX software used in the scan analysis. For subsequent weighted logistic regression models, the medians of TAFM and VATM percentages in body weight were calculated, and participants were categorized into high and low percentage groups based on medians.

### 2.3 Exposures

OBS was initially established by Zhang et al. (16) and has been extensively validated and widely used in previous studies. It is composed of 16 dietary and 4 lifestyle factors (Supplementary Table S1). Dietary OBS includes fiber, carotene, riboflavin, niacin, vitamin B6, total folate, vitamin B12, vitamin C, vitamin E, calcium, magnesium, zinc, copper, selenium, total fat, and iron, and lifestyle OBS is composed of BMI, physical activity, alcohol consumption, and smoking status. According to the oxidative properties of these 20 factors, they could additionally be categorized into pro-oxidants (total fat, iron, alcohol intake, BMI, and smoking) and antioxidants (the other 15 components).

Dietary nutrient intakes and alcohol consumption were calculated with the mean of 2 24-h dietary recalls. BMI was calculated as weight in kilograms divided by height in meters squared, and smoking status was assessed by serum cotinine with an isotope-dilution high-performance liquid chromatography/atmospheric pressure chemical ionization tandem mass spectrometric method. In terms of physical activity, we specifically focused on leisure-time physical activity (LTPA), which was calculated with the formula: two times vigorous physical activity (VPA) plus moderate physical activity (MPA), as 1 min of VPA was defined as equivalent to 2 min of MPA according to PA guidelines (29).

OBS components were assigned scores by sex and tertiles, in which the highest tertile was scored 2 and the lowest tertile was scored 0, while the intermediate tertile was scored 1 in antioxidants, and the scoring method in pro-oxidants was just the opposite. OBS was obtained by summing the total scores of 20 components, and a higher OBS indicates a higher level of antioxidant exposure. In addition, OBS was further categorized with quartiles, and the lowest quartile was set as the reference group for comparison in the weighted logistic regression models.

### 2.4 Assessment of covariates

Demographic variables include sex (male and female), age group (young adults, 20–39 years; middle-aged adults, 40–59 years) race (non-Hispanic White, non-Hispanic Black, Mexican Americans, and other races), education level (less than high school degree, high school degree, and more than high school degree), family income level (measured as the ratio of family income to poverty (PIR), low family income, PIR ≤ 1.3; middle family income, 1.3 < PIR < 3.5; high family income, PIR ≥ 3.5), and marital status (married or living with partner; divorced, separated, or widowed; never married). Additionally, total energy intake (expressed as kilocalories) and an array of chronic non-communicable diseases, including hypertension, cardiovascular diseases, and diabetes, were included due to the associations with TAFM and VATM. Hypertension was defined as average systolic pressure ≥ 140 mm Hg and/or diastolic pressure ≥ 90 mm Hg in three tests or self-reported hypertension. Cardiovascular diseases were defined as a self-reported doctor’s diagnosis of congestive heart failure, coronary heart disease, angina, myocardial infarction, or stroke. Diabetes was defined as fasting plasma glucose ≥7.0 mmol/L, 2-h plasma glucose ≥11.0 mmol/L, hemoglobin A1c ≥ 6.5%, or self-reported diabetes by a professional doctor (27, 30–32).

### 2.5 Statistical analysis

Concerning the complex sampling design in NHANES, dietary 2-day sample weight, clustering, and stratification were taken into account. Dietary 2-day sample weight divided by 4 was utilized to ensure the current study results are representative of the national population on account of the combination of four consecutive cycles.

Continuous variables were expressed as weighted means (standard deviations), and categorical variables were presented with numbers (weighted percentages) in characteristic descriptions. To compare the characteristics across quartiles, analyses of variance and Rao-Scott adjusted chi-square tests were used to test the differences in characteristics between adults with different OBS quartiles.

Both univariate and multivariate weighted logistic regression models were employed to explore the relationships between OBS and TAFM with VATM percentages in the general population. Model 1 was adjusted for demographic data (sex, age group, race, education level, income level, and marital status), and Model 2 was additionally adjusted for healthy eating index-2015 (HEI-2015) and total energy intake, while Model 3 further expanded the adjustments of disease conditions (hypertension, cardiovascular diseases, and diabetes) based on Model 2. Trend tests (p for trend) were performed by entering the quartile-categorical OBS as a continuous variable and rerunning the corresponding regression models. Three sensitivity analyses were further conducted to validate the robustness of our results: (1) missing values except for TAFM, VATM, and OBS were interpolated using the random forest interpolation methodology to reduce the potential selection bias induced by missing values in other variables; (2) we explored the immediate correlations of OBS with high TAFM and VATM without converting TAFM and VATM into percentages; and (3) we replaced BMI by sedentary duration as one of the lifestyle factors to build a revised OBS considering the drastic impact of BMI on TAFM and VATM percentages. Stratified analyses were conducted to investigate whether the associations differ by demographic variables (gender, age, race, education level, income level, and marital status), and interaction effects were tested with a likelihood test. Moreover, dietary OBS and lifestyle OBS were separately employed to assess the associations between TAFM and VATM percentages. Weighted restricted cubic spline analyses were utilized to examine the non-linear correlations of OBS with TAFM and VATM percentages, while non-linearity was assessed using the Wald test.

Stata software (version 17.0, StataCorp LLC) was utilized for most statistical analyses except for analyses of variance in R. All statistical tests were two-sided, and significance was considered at *α* = 0.05.

## 3 Results

### 3.1 Characteristics

Characteristics of adults grouped by quartiles of OBS are presented in Table 1. The final sample included 8,734 young and middle-aged adults representative of 134.7 million non-institutionalized U.S. adults (4,291 [weighted 50.9%] men; 3,299 [weighted 62.3%] non-Hispanic white; mean [SD] TAFM percentage, 2.53% [0.85%]; mean [SD] VATM percentage, 0.59% [0.26%]). Meanwhile, the medians of TAFM and VATM percentages were 2.53 and 0.553%, respectively.

**Table 1:** Characteristics by quartiles of the OBS.

Characteristics	Overall (*N* = 8,734)	Q1 (2–14) (*n* = 2,477)	Q2 (15–20) (*n* = 2,173)	Q3 (21–26) (*n* = 2,181)	Q4 (27–37) (*n* = 1903)	*p*-value
*Sex* (*n*/%)						0.2615
Male	4,291 (50.9%)	1,193 (49.3%)	1,093 (53.9%)	1,069 (50.2%)	936 (50.5%)	
Female	4,443 (49.1%)	1,284 (50.7%)	1,080 (46.1%)	1,112 (49.8%)	967 (49.5%)	
*Age group* (*n*/%)						0.2481
Young adults (20–39 years)	4,259 (49.1%)	1,179 (48.1%)	1,078 (48.9%)	1,029 (48.2%)	973 (51.4%)	
Middle-aged adults (40–59 years)	4,475 (50.9%)	1,298 (51.9%)	1,095 (51.1%)	1,152 (51.8%)	930 (48.6%)	
*Race* (n/%)						<0.0001
Non-Hispanic white	3,299 (62.3%)	914 (58.0%)	810 (61.2%)	816 (62.9%)	759 (67.4%)	
Non-Hispanic Black	1895 (11.2%)	774 (18.5%)	482 (11.6%)	372 (8.5%)	267 (6.0%)	
Mexican Americans	1,177 (9.9%)	253 (8.6%)	295 (10.3%)	328 (10.4%)	301 (10.6%)	
Other races	2,363 (16.6%)	536 (14.9%)	586 (17.0%)	665 (18.2%)	576 (16.1%)	
*Education level* (*n*/%)						<0.0001
< High school	1,397 (11.3%)	512 (16.4%)	365 (12.4%)	299 (9.8%)	221 (6.6%)	
High school	1871 (21.9%)	679 (29.9%)	453 (23.3%)	452 (21.2%)	287 (13.0%)	
>High school	5,466 (66.7%)	1,286 (53.8%)	1,355 (64.3%)	1,430 (69.0%)	1,395 (80.4%)	
*Family income level* (n/%)						<0.0001
Low family income	2,794 (23.6%)	1,008 (33.4%)	723 (24.1%)	585 (20.0%)	478 (16.5%)	
Medium family income	3,114 (33.8%)	924 (35.9%)	763 (35.0%)	776 (33.9%)	651 (30.2%)	
High family income	2,826 (42.7%)	545 (30.8%)	687 (41.0%)	820 (46.2%)	774 (53.2%)	
*Marital Status* (*n*/%)						0.0004
Married or living with a partner	5,278 (62.2%)	1,360 (56.2%)	1,304 (62.5%)	1,391 (63.7%)	1,223 (66.9%)	
Divorced, separated, or widowed	1,345 (13.4%)	433 (16.2%)	308 (14.5%)	290 (12.6%)	214 (10.2%)	
Never married	2,211 (24.4%)	684 (27.6%)	561 (23.1%)	500 (23.8%)	466 (22.9%)	
*Hypertension* (n/%)	2,998 (31.7%)	1,009 (37.5%)	725 (31.4%)	731 (30.6%)	533 (26.9%)	0.0001
*Cardiovascular diseases* (n/%)	357 (3.5%)	155 (5.1%)	74 (2.3%)	84 (4.7%)	44 (1.6%)	<0.0001
*Diabetes* (n/%)	974 (8.9%)	341 (12.4%)	262 (8.3%)	214 (8.9%)	157 (5.6%)	<0.0001
*Total energy intake (kcal), Mean (SD)*	2165.8 (832.1)	1586.8 (556.3)	2032.7 (618.7)	2330.2 (702.5)	2732.1 (947.1)	<0.001
*HEI-2015, Mean (SD)*	52.92 (13.5)	45.58 (11.28)	50.66 (12.28)	55.31 (12.95)	60.33 (12.75)	<0.001
*TAFM percentage (%), Mean (SD)*	2.53 (0.85)	2.74 (0.86)	2.55 (0.82)	2.52 (0.85)	2.29 (0.83)	<0.001
*VATM percentage (%), Mean (SD)*	0.59 (0.26)	0.64 (0.27)	0.61 (0.26)	0.59 (0.26)	0.52 (0.25)	<0.001

HEI-2015, healthy eating index-2015; TAFM, total abdominal fat mass; VATM, visceral adipose tissue mass.

In comparison to the lowest OBS quartile, adults in the higher quartiles were more likely to be non-Hispanic white, married or living with a partner, less likely to be comorbid with hypertension, cardiovascular diseases and diabetes, had a higher education level, family income, HEI-2015 scores, and total energy intake, and had lower TAFM and VATM percentages. Nevertheless, no significant differences in sex and age groups were observed across quartiles.

### 3.2 Associations between OBS and TAFM with VATM percentages

As described in Table 2, weighted logistic regression analyses revealed the negative association of continuous OBS and OBS quartiles with TAFM and VATM percentages. Compared with the lowest quartile of OBS, the second (OR: 0.70, 95%CI: 0.53–0.94), the third (OR: 0.49, 95%CI: 0.36–0.60), and the highest quartiles (OR: 0.25, 95%CI: 0.18–0.36) of OBS were associated with lower risks of high TAFM percentage in the fully adjusted model (Model 3). Similarly, the third (OR: 0.70, 95%CI: 0.54–0.92) and highest quartiles (OR: 0.56, 95%CI: 0.41–0.77) of OBS were associated with lower risks of high VATM percentage in Model 3 in comparison to the lowest quartile of OBS, while no significant differences were observed between the second quartile and the lowest quartile of OBS.

**Table 2:** Relationships between OBS and TAFM with VATM percentages in the general population.

OBS	High total abdominal fat mass percentage (>2.53%)			High visceral adipose tissue percentage (>0.553%)		
	Model 1	Model 2	Model 3	Model 1	Model 2	Model 3
Continuous	0.936(0.925,0.948)	0.920(0.903,0.937)	0.925(0.908,0.942)	0.961(0.944,0.978)	0.956(0.939,0.973)	0.963(0.954,0.973)
Q1	Ref	Ref	Ref	Ref	Ref	Ref
Q2	0.71 (0.54,0.94)	0.66 (0.49,0.88)	0.70 (0.53,0.94)	0.95 (0.75,1.21)	0.93 (0.73,1.18)	0.97 (0.79,1.28)
Q3	0.53 (0.41,0.68)	0.46 (0.33,0.62)	0.49 (0.36,0.66)	0.68 (0.56,0.84)	0.66 (0.50,0.87)	0.70 (0.54,0.92)
Q4	0.28 (0.21,0.37)	0.23 (0.16,0.32)	0.25 (0.18,0.36)	0.53 (0.43,0.65)	0.50 (0.36,0.69)	0.56 (0.41,0.77)
*P* for trend	<0.001	<0.001	<0.001	<0.001	<0.001	<0.001

Model 1 was adjusted for demographic data (sex, age group, race, education level, income level, and marital status). Model 2 was adjusted for demographic data (sex, age group, race, education level, income level, and marital status), total energy intake, and HEI-2015. Model 3 was adjusted for demographic data, total energy intake, HEI-2015, and disease conditions (hypertension, cardiovascular diseases, and diabetes).

Moreover, all three sensitivity analyses showed similar correlations and trends in Table 3, indicating the robustness of the associations. To be more specific, the second, third, and highest OBS quartiles were associated with a reduced risk of high TAFM percentage, and the third and highest quartiles were related to a lower risk of high VATM percentage, while no protective role of the second quartile of OBS against high VATM percentage was exhibited.

**Table 3:** Relationships between OBS and TAFM with VATM percentages in sensitivity analyses.

OBS	High total abdominal fat mass percentage			High visceral adipose tissue percentage		
	Model 1	Model 2	Model 3	Model 1	Model 2	Model 3
*Sensitivity analysis 1 (random forest imputation)*						
Continuous	0.936 (0.924,0.947)	0.921(0.904,0.937)	0.925(0.909,0.942)	0.962(0.953,0.972)	0.954(0.937,0.970)	0.958(0.942,0.975)
Q1	ref	ref	ref	ref	ref	ref
Q2	0.72 (0.55,0.95)	0.67 (0.51,0.89)	0.71 (0.54,0.94)	0.94 (0.75,1.19)	0.92 (0.72,1.16)	0.97 (0.76,1.23)
Q3	0.52 (0.40,0.67)	0.46 (0.34,0.61)	0.48 (0.36,0.65)	0.68 (0.55,0.84)	0.65 (0.50,0.85)	0.68 (0.53,0.89)
Q4	0.28 (0.21,0.37)	0.23 (0.16,0.33)	0.25 (0.18,0.36)	0.51 (0.42,0.63)	0.48 (0.35,0.65)	0.52 (0.38,0.72)
*P* for trend	<0.001	<0.001	<0.001	<0.001	<0.001	<0.001
*Sensitivity analysis 2 (TAFM and VATM)*						
Continuous	0.943(0.933,0.952)	0.923(0.907,0.939)	0.928(0.912,0.944)	0.953(0.944,0.962)	0.938(0.921,0.956)	0.944(0.926,0.962)
Q1	ref	ref	ref	ref	ref	ref
Q2	0.67 (0.54,0.83)	0.62 (0.49,0.78)	0.66 (0.52,0.84)	0.80 (0.65,0.99)	0.76 (0.60,0.97)	0.83 (0.65,1.07)
Q3	0.55 (0.46,0.67)	0.48 (0.37,0.62)	0.51 (0.39,0.66)	0.60 (0.49,0.73)	0.55 (0.41,0.74)	0.68 (0.54,0.87)
Q4	0.34 (0.26,0.43)	0.27 (0.19,0.39)	0.30 (0.21,0.44)	0.44 (0.36,0.55)	0.39 (0.27,0.56)	0.47 (0.37,0.61)
*P* for trend	<0.001	<0.001	<0.001	<0.001	<0.001	<0.001
*Sensitivity analysis 3 (revised OBS)*						
Continuous	0.938(0.927,0.950)	0.924(0.907,0.941)	0.928(0.910,0.945)	0.967(0.957,0.976)	0.962(0.945,0.979)	0.967(0.950,0.983)
Q1	ref	ref	ref	ref	ref	ref
Q2	0.69 (0.54,0.89)	0.65 (0.50,0.85)	0.69 (0.53,0.89)	0.89 (0.72,1.11)	0.88 (0.71,1.10)	0.95 (0.75,1.19)
Q3	0.50 (0.39,0.62)	0.56 (0.45,0.70)	0.49 (0.36,0.61)	0.70 (0.57,0.86)	0.68 (0.52,0.90)	0.73 (0.56,0.95)
Q4	0.30 (0.23,0.39)	0.40 (0.30,0.52)	0.28 (0.20,0.39)	0.55 (0.45,0.67)	0.53 (0.38,0.74)	0.58 (0.42,0.82)
*P* for trend	<0.001	<0.001	<0.001	<0.001	<0.001	0.001

Model 1 was adjusted for demographic data (sex, age group, race, education level, income level, and marital status). Model 2 was adjusted for demographic data (sex, age group, race, education level, income level, and marital status), total energy intake, and HEI-2015. Model 3 was adjusted for demographic data (sex, age group, race, education level, income level, and marital status), total energy intake, HEI-2015, and disease conditions (hypertension, cardiovascular diseases, and diabetes).

### 3.3 Subgroup analyses and interaction effects of the associations between OBS and TAFM with VATM percentages

Tables 4, 5 exhibit the associations between OBS and TAFM with VATM percentages in demographic subpopulations in the fully adjusted models. Higher OBS quartiles were correlated with lower risks of high TAFM percentage in all subgroups, and we observed the trends in ORs across OBS quartiles except for adults with high school education level. In addition, significant interaction effects of education (*p* = 0.0390) and income (*p* = 0.0031) on the relationships between OBS and the risks of a high TAFM percentage were discovered, while no other interactions were inferred. Higher OBS quartiles were associated with lower risks of high VATM percentage in various demographic subpopulations, and the trends across OBS quartiles were not present in Mexican Americans, adults with less than or equal to high school education level, adults with low and medium family income, and divorced, separated, or widowed and never married adults. Furthermore, significant interaction effects of sex (*p* = 0.0198) and education (*p* = 0.0423) were shown, manifesting as the ORs in male adults and adults with higher than high school education levels being the smallest in corresponding subpopulations.

**Table 4:** Relationships between OBS and TAFM percentage in demographic subgroups.

Characteristics	Q1	Q2	Q3	Q4	*P* for interaction	*P* for trend
*Sex*					0.0906	
Male	Ref	0.73 (0.51,1.05)	0.48 (0.32,0.73)	0.29 (0.18,0.47)		<0.001
Female	Ref	0.65 (0.43,0.98)	0.49 (0.31,0.77)	0.24 (0.14,0.42)		<0.001
*Age group*					0.1087	
Young adults (20–39 years)	Ref	0.59 (0.42,0.83)	0.42 (0.31,0.57)	0.17 (0.11,0.27)		<0.001
Middle-aged adults (40–59 years)	Ref	0.83 (0.55,1.26)	0.55 (0.34,0.91)	0.36 (0.23,0.58)		<0.001
*Race*					0.9607	
Non-Hispanic white	Ref	0.62 (0.40,0.96)	0.41 (0.25,0.67)	0.21 (0.12,0.37)		<0.001
Non-Hispanic Black	Ref	0.67 (0.42,1.08)	0.55 (0.33,0.90)	0.26 (0.13,0.55)		0.001
Mexican Americans	Ref	0.78 (0.43,1.45)	0.46 (0.23,0.92)	0.27 (0.11,0.68)		0.003
Other races	Ref	0.74 (0.49,1.14)	0.55 (0.35,0.86)	0.23 (0.12,0.42)		<0.001
*Education level*					0.0390	
< High school	Ref	0.58 (0.34,0.99)	0.63 (0.34,1.19)	0.38 (0.16,0.87)		0.039
High school	Ref	0.75 (0.44,1.30)	0.82 (0.47,1.44)	0.44 (0.21,0.92)		0.078
>High school	Ref	0.69 (0.47,1.01)	0.39 (0.26,0.58)	0.21 (0.14,0.32)		<0.001
*Income level*					0.0031	
Low family income	Ref	0.78 (0.52,1.16)	0.61 (0.40,0.93)	0.53 (0.30,0.93)		0.017
Medium family income	Ref	0.75 (0.47,1.21)	0.69 (0.43,1.12)	0.29 (0.14,0.60)		0.003
High family income	Ref	0.57 (0.35,0.93)	0.30 (0.19,0.48)	0.15 (0.09,0.25)		<0.001
*Marital status*					0.7217	
Married or living with partner	Ref	0.66 (0.45,0.95)	0.47 (0.32,0.68)	0.25 (0.16,0.40)		<0.001
Divorced, separated, or widowed	Ref	0.97 (0.48,1.98)	0.61 (0.31,1.23)	0.31 (0.13,0.75)		0.008
Never married	Ref	0.65 (0.43,0.98)	0.44 (0.27,0.72)	0.18 (0.10,0.32)		<0.001

Model 3 was adopted and adjusted for demographic data (sex, age group, race, education level, income level, and marital status), total energy intake, HEI-2015, and disease conditions (hypertension, cardiovascular diseases, and diabetes).

**Table 5:** Relationships between OBS and VATM percentage in demographic subgroups.

Characteristics	Q1	Q2	Q3	Q4	*P* for interaction	*P* for trend
*Sex*					0.0198	
Male	Ref	1.20 (0.88,1.62)	0.65 (0.45,0.93)	0.55 (0.34,0.89)		0.002
Female	Ref	0.82 (0.59,1.14)	0.76 (0.54,1.06)	0.58 (0.39,0.85)		0.012
*Age group*					0.8082	
Young adults (20–39 years)	Ref	0.91 (0.69,1.19)	0.67 (0.49,0.92)	0.49 (0.34,0.72)		<0.001
Middle-aged adults (40–59 years)	Ref	1.06 (0.74,1.52)	0.72 (0.46,1.11)	0.61 (0.38,0.99)		0.023
*Race*					0.9197	
Non-Hispanic white	Ref	0.86 (0.59,1.24)	0.50 (0.33,0.75)	0.37 (0.22,0.60)		<0.001
Non-Hispanic Black	Ref	0.71 (0.52,0.97)	0.67 (0.41,1.12)	0.53 (0.29,0.98)		0.039
Mexican Americans	Ref	1.04 (0.56,1.92)	0.74 (0.37,1.52)	0.71 (0.31,1.64)		0.321
Other races	Ref	1.03 (0.67,1.58)	0.76 (0.46,1.25)	0.56 (0.31,1.02)		0.032
*Education level*					0.0423	
< High school	Ref	1.50 (0.88,2.55)	1.17 (0.69,2.00)	1.04 (0.48,2.27)		0.851
High school	Ref	0.93 (0.64,1.36)	0.96 (0.57,1.62)	1.04 (0.48,2.26)		0.967
>High school	Ref	0.90 (0.68,1.19)	0.54 (0.39,0.75)	0.42 (0.29,0.59)		<0.001
*Income level*					0.8575	
Low family income	Ref	1.10 (0.78,1.54)	0.88 (0.62,1.26)	0.89 (0.55,1.45)		0.481
Medium family income	Ref	1.02 (0.69,1.41)	0.90 (0.57,1.41)	0.84 (0.49,1.45)		0.477
High family income	Ref	0.90 (0.59,1.38)	0.47 (0.29,0.76)	0.32 (0.20,0.53)		<0.001
*Marital status*					0.3017	
Married or living with partner	Ref	0.99 (0.74,1.32)	0.67 (0.47,0.96)	0.54 (0.36,0.81)		0.001
Divorced, separated, or widowed	Ref	1.15 (0.36,1.35)	0.70 (0.36,1.35)	0.57 (0.25,1.31)		0.128
Never married	Ref	0.84 (0.51,1.41)	0.77 (0.50,1.19)	0.56 (0.30,1.07)		0.083

Model 3 was adopted and adjusted for demographic data (sex, age group, race, education level, income level, and marital status), total energy intake, HEI-2015, and disease conditions (hypertension, cardiovascular diseases, and diabetes).

### 3.4 Associations between dietary OBS and lifestyle OBS with TAFM and VATM percentages

To evaluate the individual protective effects of dietary OBS and lifestyle OBS separately, weighted logistic regression models were employed, and the results are shown in Table 6. When compared with the lowest quartile of dietary OBS in the fully adjusted model, both the third (OR: 0.71, 95%CI: 0.55–0.93) and the highest (OR: 0.59, 95%CI: 0.41–0.84) quartiles of dietary OBS were associated with lower risks of high TAFM percentage in Model 3, with the trends of ORs across OBS quartiles being observed. Meanwhile, the second (OR: 0.60, 95%CI: 0.47–0.76), the third (OR: 0.39, 95%CI: 0.29–0.51), and the highest (OR: 0.15, 95%CI: 0.11–0.18) quartiles of lifestyle OBS were strongly negatively associated with lower risks of high TAFM percentage.

**Table 6:** Associations of dietary and lifestyle OBS with TAFM and VATM percentages.

OBS	High total abdominal fat mass percentage			High visceral adipose tissue mass percentage		
	Model 1	Model 2	Model 3	Model 1	Model 2	Model 3
*Dietary OBS*						
Continuous	0.958(0.946,0.969)	0.962(0.943,0.982)	0.964(0.945,0.984)	0.978(0.968,0.988)	0.985(0.9661.005)	0.987(0.968,1.006)
Q1 (1–10)	ref	ref	ref	ref	ref	ref
Q2 (11–16)	0.89 (0.72,1.09)	0.96 (0.76,1.20)	0.99 (0.80,1.24)	0.99 (0.83,1.17)	0.88 (0.73,1.07)	0.94 (0.78,1.15)
Q3 (17–22)	0.61 (0.49,0.77)	0.69 (0.52,0.91)	0.71 (0.55,0.93)	0.73 (0.60,0.90)	0.63 (0.49,0.82)	0.66 (0.51,0.85)
Q4 (23–31)	0.48 (0.38,0.61)	0.57 (0.40,0.83)	0.59 (0.41,0.84)	0.69 (0.57,0.93)	0.55 (0.42,0.73)	0.56 (0.42,0.73)
*P* for trend	<0.001	0.001	0.001	<0.001	<0.001	<0.001
*Lifestyle OBS*						
Continuous	0.611(0.578,0.646)	0.613(0.579,0.650)	0.634(0.599.,0670)	0.730(0.692,0.769)	0.736(0.697,0.777)	0.761(0.720,0.804)
Q1 (1–3)	ref	ref	ref	ref	ref	ref
Q2 (4)	0.57 (0.45,0.71)	0.56 (0.45,0.71)	0.60 (0.47,0.76)	0.75 (0.63,0.88)	0.75 (0.63,0.89)	0.79 (0.67,0.94)
Q3 (5)	0.35 (0.26,0.46)	0.36 (0.27,0.47)	0.39 (0.29,0.51)	0.61 (0.50,0.74)	0.64 (0.53,0.77)	0.69 (0.57,0.84)
Q4 (6–8)	0.12 (0.10,0.15)	0.13 (0.10,0.16)	0.15 (0.11,0.18)	0.25 (0.20,0.31)	0.26 (0.21,0.32)	0.30 (0.24,0.38)
*P* for trend	<0.001	<0.001	<0.001	<0.001	<0.001	<0.001

Model 1 was adjusted for demographic data (sex, age group, race, education level, income level, and marital status). Model 2 was adjusted for demographic data (sex, age group, race, education level, income level, and marital status), total energy intake, and HEI-2015. Model 3 was adjusted for demographic data, total energy intake, HEI-2015, and disease conditions (hypertension, cardiovascular diseases, and diabetes).

In comparison to adults of the lowest quartile of dietary OBS in Model 3, adults of the third (OR: 0.66, 95%CI: 0.51–0.85) and highest (OR: 0.56, 95%CI: 0.42–0.73) quartiles had 34 and 44% reduced risks of high VATM percentage, while no such effect was observed in adults in the second quartile. Apart from this, adults of the second (OR: 0.79, 95%CI: 0.67–0.94), the third (OR: 0.69, 95%CI: 0.57–0.84), and the highest (OR: 0.30, 95%CI: 0.24–0.38) quartiles of lifestyle OBS had 21, 31, and 70% reduced risks, respectively.

### 3.5 Non-linear associations between OBS, dietary OBS, and lifestyle OBS with TAFM and VATM

**Figure 2:**
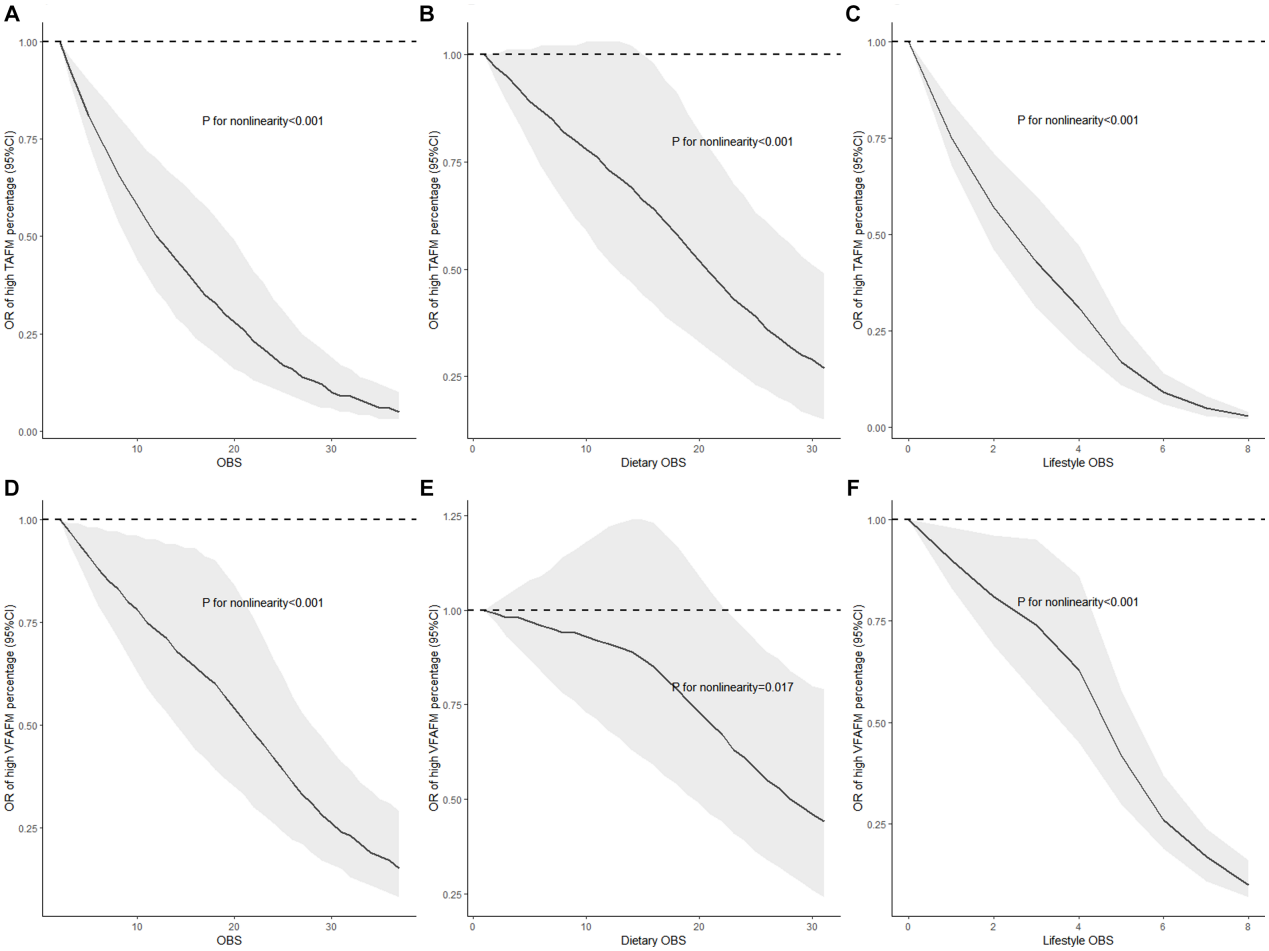
Non-linear associations of OBS, dietary OBS, and lifestyle OBS with the risks of high TAFM and VATM with restricted cubic splines. **(A)** OBS and TAFM percentage; **(B)** dietary OBS and TAFM percentage; **(C)** lifestyle OBS and TAFM percentage; **(D)** OBS and VATM percentage; **(E)** dietary OBS and VATM percentage; and **(F)** lifestyle OBS and VATM percentage. Models were fully adjusted for demographic data (sex, age group, race, education level, income level, and marital status), total energy intake, HEI-2015, and disease conditions (hypertension, cardiovascular diseases, and diabetes); TAFM, total abdominal fat mass; VATM, visceral adipose tissue mass.

Weighted restricted cubic splines were conducted to assess the non-linear associations of OBS, dietary OBS, and lifestyle OBS with the risks of high TAFM and VATM percentages, the results of which are displayed in Figure 2. Figures 2A–C show a significant non-linear correlation of OBS, dietary OBS, and lifestyle OBS with TAFM percentage, with higher OBS reflecting lower ORs. Similarly, the significant non-linear relationship between OBS, dietary OBS, and lifestyle OBS with VATM percentage is demonstrated in Figures 2D–F. The results suggest that as OBS, dietary OBS, and lifestyle OBS increase, the risks of high TAFM and VATM percentages decrease in a non-linear manner. Meanwhile, it must be pointed out that the protective role of dietary OBS against TAFM and VATM percentages was not significant when it was relatively low or medium.

## 4 Discussions

With the large cross-sectional survey, we found that OBS, dietary OBS, and lifestyle OBS were all strongly negatively associated with the risks of high TAFM and VATM percentages in U.S. young and middle-aged adults, and the results of three sensitivity analyses strengthened the robustness of the results. Moreover, the protective role of OBS against TAFM and VATM percentages was consistent in various demographic subgroups, and weighted restricted cubic splines revealed that the higher the OBS, dietary OBS, and lifestyle OBS, the lower the risks of high TAFM and VATM percentages.

OBS, based on dietary and lifestyle factors but not endogenous antioxidants or pro-oxidants, was adopted in our study to speculate on the individual oxidative stress level. OBS was proven to be positively related to leukocyte telomere length in females when it was developed, while telomere length was reported by multiple experiments and reviews to be correlated with oxidative stress (33–35). In addition, the associations of OBS with oxidative stress were verified in a study that assessed the mediation role of oxidative stress between OBS and cognitive function, as well as another study of the mediation role of oxidative stress between OBS and depressive symptoms (17, 18). Indeed, the original OBS was first developed in 2002, and it only included vitamin C, beta-carotene, and iron (36). With the deepening of research on antioxidants and oxidants, OBS was expanded and updated, considering part or all of dietary, biomarkers, lifestyle, and medication to include a wider range of factors in relation to oxidative stress, while the OBS utilized in our study was based on NHANES data, in which sex-specific differences were taken into account (37).

Numerous studies have explored the association between oxidative stress and obesity, and most researchers have approved the correlation, similar to our results. Marseglia L found an inverse association between body fat, central adiposity, and serum total antioxidant capacity in a population-based study (38). Moreover, a study of 2,367 Korean adults found a positive relationship between abdominal obesity and oxidative stress measured by derivatives of reactive oxygen metabolite concentration, in which abdominal obesity was thought to increase oxidative stress and affect the signaling pathways involved in obesity (39). Nevertheless, studies about visceral fat accumulation were relatively rare. A cross-sectional study found a relationship between visceral obesity and lipoperoxidation with oxidative DNA damage but not endogenous antioxidant defenses (40).

In addition, the associations of OBS with TAFM and VATM percentages were robust in demographic subpopulations, and the ORs of VATM in male adults were lower, indicating a stronger protective effect of OBS in men. Women were found to possess a lower oxidative stress level, lower oxidative stress biomarkers, lower reactive oxygen species production, and greater antioxidant potential, suggesting that women were less likely to be susceptible to oxidative stress (41–45). Meanwhile, women are more resistant than men to several diseases involving oxidative stress, and cell-autonomous mechanisms also contribute to the resistance of female cells to oxidative stress-induced apoptosis, suggesting that women have a higher ability to handle oxidative stress (46). Specifically, men have a higher oxidative stress level than women, and the reduction of oxidative stress levels across quartiles of OBS in men is greater than that in women. As a result, stronger changes in oxidative stress levels across quartiles in men lead to greater decreasing ORs, showing a stronger protective effect of OBS in men. Nevertheless, more studies are required to validate our sex-specific hypothesis and explore whether the interaction effects between education level and TAFM and VATM are accidental.

Some dietary nutrients, such as fiber, folate, vitamin C, total fats, calcium, magnesium, and copper, were thought to reduce the obesity risk, but more studies focused on serum levels, while dietary selenium intake was not shown to be correlated with obesity (47–53). Nevertheless, a population-based ecological study concluded that long-term exposure to high levels of B vitamins may be involved in the increased prevalence of obesity in the United States (54). Iron deficiency was found to be positively correlated with obesity, and iron metabolism was altered in obese people, even if iron promoted oxidative stress as a pro-oxidant (55, 56). Consequently, in-depth mechanism studies about the complex signaling pathways of iron metabolism, oxidative stress, and obesity were urgently required. Lifestyle OBS, consisting of physical activity, alcohol intake, BMI, and cotinine, was a platitude related to obesity (57). We replaced BMI with sedentary duration to eliminate the exceptionally strong effect of BMI, and the association persisted. Based on previous research, sedentary duration may also be a lifestyle indicator of oxidative stress (58). Furthermore, non-linear correlations between OBS, dietary OBS, and lifestyle OBS with risks of high TAFM and VATM percentages were exhibited in weighted restricted cubic splines, and the relationships between dietary OBS and the risks of high TAFM and VATM percentages were not linear and did not exist in adults with low and medium dietary OBS, suggesting that the protective effects may vary at different levels of dietary OBS and adults should maintain at relatively higher dietary OBS to reduce the risks of high TAFM and VATM percentages.

The findings underline the significance of adhering to an antioxidant diet and lifestyle in reducing the risks of high TAFM and VATM percentages indicating abdominal obesity and visceral fat accumulation, and it is crucial to make the adults aware of the strong association and thus encourage them to provide possible preventive measures and make necessary changes.

The major strength of this study is the use of a large, nationally representative U.S. survey and the combination of data in four cycles, increasing the sample size and enlarging the generalizability of our results. Furthermore, the adoption of subgroup analyses, interaction effects, and sensitivity analyses enhances the robustness and credibility of our results. Finally, restricted cubic splines demonstrated significant non-linear dose–response relationships, which were more intuitive. Nevertheless, it cannot be denied that our study also has some limitations. First, only associations rather than causality could be inferred from this study since NHANES were cross-sectional studies, and further longitudinal or interventional studies are required to better understand the causal relationship. Second, more prospective and high-quality studies are urgently needed to evaluate the effectiveness of OBS. Additionally, the heterogeneity in the definition of an OBS results in contradictory results among OBS studies for some outcomes. Consequently, some covariates were not based on clinical data but self-reported, the reliability of which is relatively weak. Finally, quartiles of OBS were used in our study population, weakening its comparability to other studies with different cut-points.

## Data availability statement

The raw data supporting the conclusions of this article will be made available by the authors under reasonable requirements, without undue reservation.

## Author contributions

KW: Data curation, Formal analysis, Methodology, Software, Supervision, Validation, Visualization, Writing – original draft. MD: Data curation, Formal analysis, Writing – review & editing. JW: Writing – review & editing. LL: Writing – review & editing. RC: Conceptualization, Methodology, Writing – review & editing. FL: Writing – review & editing. JN: Data curation, Formal analysis, Writing – review & editing. FT: Writing – review & editing. QL: Writing – review & editing. XL: Writing – review & editing. FX: Writing – review & editing.

## Footnotes

^1^ https://www.cdc.gov/nchs/nhanes/index.htm
