# Supplementary material for: Associations of oxidative balance score with total abdominal fat mass and visceral adipose tissue mass percentages among young and middle-aged adults: findings from NHANES 2011–2018
Source: Front Nutr. 2023 Dec 5;10:1306428. doi: 10.3389/fnut.2023.1306428 (PMC10728272; doi:10.3389/fnut.2023.1306428)
Supplement: Supplementary file 1 [file Table_1.DOCX]

Supplementary Material

**Table S1 Components of the OBS**

| OBS components | Property | Male | | | Female | | |
| --- | --- | --- | --- | --- | --- | --- | --- |
|  |  | 0 | 1 | 2 | 0 | 1 | 2 |
| Dietary OBS components | | | | | | | |
| Dietary fiber (g/d) | A | <13.90 | 13.90-21.55 | ≥21.55 | <11.20 | 11.20-17.70 | ≥17.70 |
| Carotene (RE/d) | A | <141.42 | 141.42-577.50 | ≥577.50 | <158.25 | 158.25-662.33 | ≥662.33 |
| Riboflavin (mg/d) | A | <1.86 | 1.86-2.71 | ≥2.71 | <1.43 | 1.43-2.08 | ≥2.08 |
| Niacin (mg/d) | A | <25.15 | 25.15-34.75 | ≥34.75 | <17.40 | 17.40-17.40 | ≥24.66 |
| Vitamin B_6_ (mg/d) | A | <1.89 | 1.89-2.77 | ≥2.77 | <1.37 | 1.37-2.00 | ≥2.00 |
| Total folate (mcg/d) | A | <343.50 | 343.50-508.00 | ≥508.00 | <263.50 | 263.50-397.50 | ≥397.50 |
| Vitamin B_12_ (mcg/d) | A | <3.63 | 3.63-6.31 | ≥6.31 | <2.61 | 2.61-4.51 | ≥4.51 |
| Vitamin C (mg/d) | A | <36.65 | 36.65-92.30 | ≥92.30 | <36.50 | 36.50-83.75 | ≥83.75 |
| Vitamin E (ATE) (mg/d) | A | <7.02 | 7.02-11.12 | ≥11.12 | <5.84 | 5.84-9.23 | ≥9.23 |
| Calcium (mg/d) | A | <820.50 | 820.50-1244.0 | ≥1072.00 | <662.50 | 662.50-990.50 | ≥990.50 |
| Magnesium (mg/d) | A | <277.00 | 277.00-386.50 | ≥386.50 | <218.00 | 218.00-301.00 | ≥301.00 |
| Zinc (mg/d) | A | <10.21 | 10.21-14.69 | ≥14.69 | <7.42 | 7.42-10.68 | ≥10.68 |
| Copper (mg/d) | A | <1.06 | 1.06-1.57 | ≥1.57 | <0.85 | 0.85-1.22 | ≥1.22 |
| Selenium (mcg/d) | A | <109.75 | 109.75-152.90 | ≥152.90 | <79.55 | 79.55-111.00 | ≥111.00 |
| Total fat (g/d) | P | ≥106.71 | 75.86-106.71 | <75.86 | ≥80.72 | 56.10-80.72 | <56.10 |
| Iron (mg/d) | P | ≥18.31 | 12.72-18.31 | <12.72 | ≥14.04 | 9.79-14.04 | <9.79 |
| Lifestyle OBS components | | | | |  |  |  |
| Physical activity (LTPA) | A | 0 | 0-300 | ≥300 | 0 | 0-225 | ≥225 |
| Alcohol intake (g/d) | P | ≥30 | 0-30 | 0 | ≥15 | 0-15 | 0 |
| Body mass index (kg/m^2^) | P | ≥30.50 | 25.90-30.50 | <25.90 | ≥28.64 | 23.74-28.64 | <23.74 |
| Cotinine (ng/mL) | P | ≥1.38 | 0.017-1.38 | <0.017 | ≥0.107 | 0.011-0.107 | <0.011 |

A, antioxidant; ATE, alpha-tocopherol equivalent; OBS, oxidative balance score; P, pro-oxidant; RE, retinol equivalent.
